# Public health facility resource availability and provider adherence to first antenatal guidelines in a low resource setting in Accra, Ghana

**DOI:** 10.1186/s12913-016-1747-1

**Published:** 2016-09-21

**Authors:** Mary Amoakoh-Coleman, Irene Akua Agyepong, Gbenga A. Kayode, Diederick E. Grobbee, Kerstin Klipstein-Grobusch, Evelyn K. Ansah

**Affiliations:** 1 Julius Global Health, Julius Center for Health Sciences and Primary Care, University Medical Centre, Utrecht, Netherlands; 2 School of Public Health, University of Ghana, Legon, Ghana; 3 Division of Epidemiology and Biostatistics, School of Public Health, Faculty of Health Sciences, University of the Witwatersrand, Johannesburg, South Africa; 4 Research and Development Division, Ghana Health Service, Accra, Ghana

**Keywords:** Adherence, Facility factors, First antenatal care, Guidelines, Providers, Support

## Abstract

**Background:**

Lack of resources has been identified as a reason for non-adherence to clinical guidelines. Our aim was to describe public health facility resource availability in relation to provider adherence to first antenatal visit guidelines.

**Methods:**

A cross-sectional analysis of the baseline data of a prospective cohort study on adherence to first antenatal care visit guidelines was carried out in 11 facilities in the Greater Accra Region of Ghana. Provider adherence was studied in relation to health facility resource availability such as antenatal workload for clinical staffs, routine antenatal drugs, laboratory testing, protocols, ambulance and equipment.

**Results:**

Eleven facilities comprising 6 hospitals (54.5 %), 4 polyclinics (36.4 %) and 1 health center were randomly sampled. Complete provider adherence to first antenatal guidelines for all the 946 participants was 48.1 % (95 % CI: 41.8–54.2 %), varying significantly amongst the types of facilities, with highest rate in the polyclinics. Average antenatal workload per month per clinical staff member was higher in polyclinics compared to the hospitals. All facility laboratories were able to conduct routine antenatal tests. Most routine antenatal drugs were available in all facilities except magnesium sulphate and sulphadoxine-pyrimethamine which were lacking in some. Antenatal service protocols and equipment were also available in all facilities.

**Conclusion:**

Although antenatal workload varies across different facility types in the Greater Accra region, other health facility resources that support implementation of first antenatal care guidelines are equally available in all the facilities. These factors therefore do not adequately account for the low and varying proportions of complete adherence to guidelines across facility types. Providers should be continually engaged for a better understanding of the barriers to their adherence to these guidelines.

## Background

**Fig. 1:**
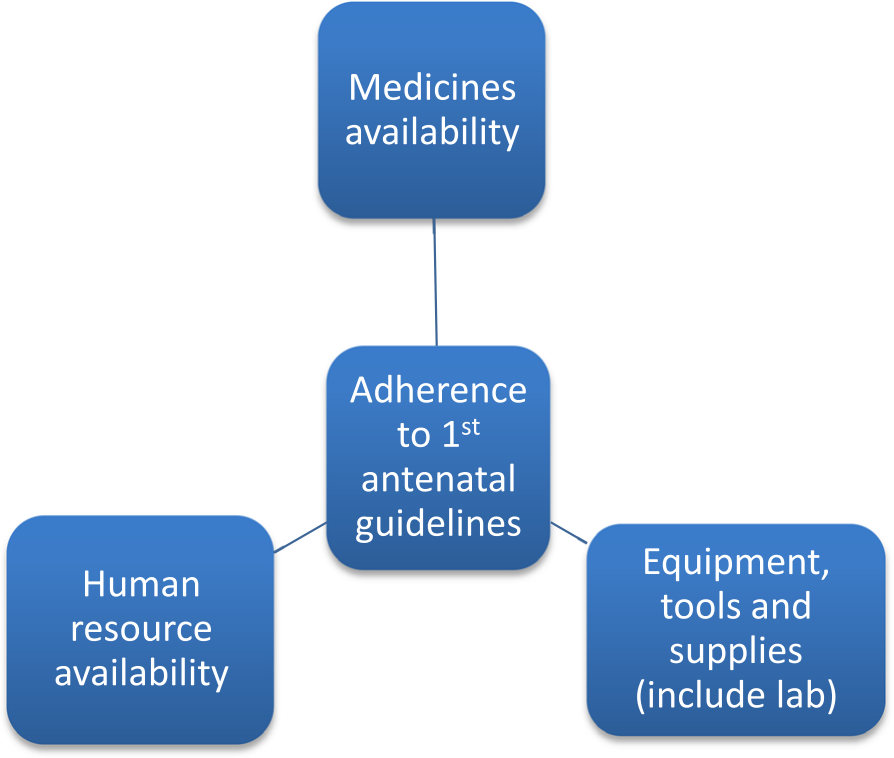
Health facility resources affecting frontline provider adherence to first antenatal guidelines. Legend: A diagrammatic presentation of the concept of health facility resources that affect frontline provider adherence to first antenatal visit guidelines

Guidelines are developed to ensure quality, uniformity and consistency of care for clients [1]. Engagement of providers during the process of developing guidelines is important in ensuring that they adhere to the guidelines [2]. Many reasons have been reported for provider non-adherence to guidelines, for example guidelines being unclear, too rigid to apply or provider disagreement with the guidelines [1, 3, 4]. These reasons also include factors external to the provider such as lack of resources for service provision [5, 6]. It is therefore important to understand the setting within which providers work and how this supports or hinders the implementation of available guidelines requirement. Adequate human resource capacity, appropriate infrastructure and equipment as well as supplies are essential to support care providers and enhance their adherence to guideline procedures. Figure 1 is an illustration of this framework.

The National Safe Motherhood Service Protocol (SMP) [7] was developed to support Ghana’s efforts in adopting the Safe Motherhood Initiative developed by the World Health Organization (WHO) [8]. The protocol contains specific requirements for first antenatal clinic attendants in terms of history to take, physical examination, laboratory tests, routine medication and counselling. Health workers have subsequently been trained to use this protocol in their daily work [9]. It is, however, known that there are varying levels of adherence to guidelines [9–11], and in Ghana the extent to which evidence based guidelines are used by frontline providers in care decision making is uncertain [9]. It is also known that there is discrepancy between provider knowledge and practice [12], especially in low and middle income settings, and reasons for this need to be explored. In a study in Ghana to understand why and how caregivers make clinical decisions for clients, health system resource constraints such as availability of staff, medicines, supplies and equipment were listed as key in influencing decisions made in service delivery [9]. Understandably, laboratory logistics need to be in place for a provider to request laboratory tests as demanded in a protocol. Routine medication for a pregnant woman needs to be available in the pharmacy for the provider who wants to prescribe them as the protocol may require.

Our aim was to describe public health facility resource availability in relation to provider adherence to first antenatal visit guidelines.

## Methods

A cross-sectional analysis of the baseline data of a prospective cohort study on adherence to guidelines was carried out. The prospective study was conducted to assess the effect of provider adherence to first antenatal guidelines on pregnancy outcomes. To better understand the adherence levels, we found it necessary to do a facility audit at baseline to assess facility resource availability.

### Study setting

The study was conducted in the Greater Accra Region (GAR) of Ghana. The region has a total of 20 health administrative districts and sub-metropolises and is served by both public and private facilities. About 97 % of pregnant women here attend antenatal clinic at least once during their pregnancy, with 91 % attending four times or more [13]. Also, 62 % of all health facility deliveries in the region take place in the public sector [13]. The public sector comprises one teaching hospital, one regional hospital and nine district and sub-metropolitan hospitals. District and sub-metropolitan hospitals are at the same level and provide the same level of care, although they may serve different population sizes. There are ten polyclinics, 31 health centers, 19 community clinics and three Community-Based Health Planning and Services (CHPS) compounds. There are 11 districts and sub-metropolises without hospitals. The hospitals usually have specialist obstetricians working there in addition to medical officers and other categories of health workers that one will find in the polyclinics. They also often have operating theaters for obstetric surgeries, an infrastructure that the polyclinics lack. The polyclinics are primary health care facilities like the health centers, providing services for different conditions in different units. They are run by non-specialist medical officers and medical or physician assistants, together with midwives, nurses and other paramedical staffs. Cases are referred from polyclinics to the district hospitals. The health centers are usually rural and may or may not have a medical officer. CHPS compounds operate at the community level with midwives or community health nurses who provide basic primary care services like health education, treatment of minor ailments and injuries, antenatal services, normal deliveries, and postnatal services. There are several private hospitals in the region. The National Health Insurance Scheme (NHIS) is operational in all the public as well as in most of the private facilities [14].

#### Sample size for prospective study

The sample size for the cohort study was based on a prevalence of pregnancy complications of 6 % [15] using Open Epi calculator [16] for estimation. We assumed that the complication rate will be twice as high amongst the exposed group (incomplete adherence). To detect a two-sided significance difference at 95 % confidence interval, at a power of 80 %, and a one-to-one ratio of exposure to non-exposure, a sample of 372 women was required per exposure group. The total sample size therefore required for both arms was 744.

### Selection of districts and facilities

We randomly selected participants from the different levels of public health care (variable “type of facility”) across the Greater Accra region. All districts and sub-metropolises in the region were grouped into those with a district or sub-metropolitan hospital (eight in number) and those without hospitals (12 in total) and then five districts were randomly selected by balloting with replacement from each group. By this process, the names of the districts in each group were written on a piece of paper, folded and put in a box. After shuffling by an independent person, five districts were picked one at a time, replacing the picked district in the box each time before picking the next to ensure equal chance of selection for all. The district hospitals in the selected districts *with* hospitals were included in our study. In those districts without hospitals all the primary level care facilities offering both antenatal and delivery services were included for random sampling and one was selected from each district.” We also included the regional hospital which is in a sub-metropolis different from the selected districts/ metropolises. Thus in all, 11 health facilities (one regional and five district/sub-metropolitan hospitals, four polyclinics and a health center) were randomly selected from 11 districts/ sub-metropolises for the study.

### Data collection processes and tools

At recruitment, a record review of participants’ first antenatal care (ANC) visit from the maternal health record book was carried out using a checklist alongside recording of data on their socio-demographic characteristics, as well as the 13 variables on guideline requirements. A facility audit was conducted to assess facility factors such as the availability of personnel, services, infrastructure, logistics and supplies that are required to support adherence to the first antenatal care guidelines at facility level. The assumption was made that any information on history, examination, laboratory examination and treatment available is what was recorded in the maternal health record book. Information on any service not recorded, was deemed not to have been delivered.

### Variables

Facility factors assessed in the current study were type of facility, antenatal workload in each facility per month, availability of blood, ambulance, routine antenatal drugs (and other essential drugs), laboratory testing required especially for first antenatal care, protocol availability and equipment. Data on challenges faced by facilities with respect to antenatal and delivery services was also collected. A facility audit tool was used to collect all the data and it involved interviewing facility managers as well as heads of antenatal, delivery, laboratory, records and pharmacy units.

Provider adherence to first antenatal guidelines was assessed by use of a 13-point checklist to score this adherence. The questions on the checklist were based on the requirements for first ANC visit as per the SMP for Ghana, which is also consistent with the national treatment guidelines for first ANC visit. Two of the variables on the checklist, herein referred to as “optional” variables, may not be due at the first ANC visit, depending on the woman’s gestational age and therefore do not influence the adherence categorization. These are the “last pregnancy history if applicable” and “Intermittent Presumptive Treatment in pregnancy (IPTp) given if woman is due”. IPTp is indicated for women in the second and third trimesters only. The remaining 11 variables are required for all clients irrespective of the gestational age (“age”, “parity”, “gestational age at booking”, “medical, surgical or family history”, “weight”, “blood pressure”, “abdomen examination”, “hemoglobin test”, “urine test”, “iron supplement”, “tetanus injection”, and are herein referred to as “mandatory” variables). Every record reviewed was assessed to see how many of the 13 variables were actually adhered to by the provider at the first ANC visit. Each variable adhered to, scored a point of 1 while non-adherence scored 0. A total score of *11*–*13* (including a score of 1 to all the 11 mandatory variables) was classified as complete adherence to guidelines. Non-adherence to any of the mandatory variables was classified as incomplete adherence. Incomplete adherence was re-categorized into *moderate* adherence (score of 11–12) and *poor* adherence (score < 11) during data analysis. Table 1 describes the variables on the checklist and the scoring criteria.

**Table 1:** General characteristics of facilities sampled

Variable	Frequency *N* (%)
Facility type	
Hospital	6 (54.5)
Polyclinic	4 (36.4)
Health center	1 (9.1)
Location	
Urban	10 (90.9)
Rural	1 (9.1)
Focused ANC	
Yes	7 (63.6)
No	2 (18.2)
Modified	2 (18.2)
Frequency of ANC services per week	
Every week day	9 (81.9)
3–4 days	2 (18.2)
Routine ANC services	11 (100.0)
Weight taken	
Blood pressure taken	
History taken	
Tetanus injection given	
Health talk given	
Client counselled	
SMP available	11 (100.0)
Clinical staff at antenatal clinic	
Doctors & midwives	11 (100.0)
Other health workers	0 (0.0)

*ANC* means antenatal care; *SMP* means national safe motherhood service protocol

#### Measuring human resource availability

The human resource capacity measures used were total and mean number of critical clinical and support staffs, average antenatal workload per month per clinical staff and workload scores (as a measure of ranking the facilities based on workload on a scale of 0–1).

Antenatal clinical staffs for antenatal care were defined as medical officers (including obstetrician-gynecologists) and midwives because all facilities indicated that only these categories of health workers attend to antenatal clients. All the clinical staffs included in our analysis work mainly in the maternity unit of the facilities. Although these staffs may provide delivery services in addition to antenatal services it is not easily possible to determine how much time each staff in the maternity unit spends on either antenatal or delivery services. We thus included all of them as clinical staffs for our purpose.

Support staffs were defined as pharmacists, pharmacy technologists, dispensing technicians, biomedical scientists, laboratory technologists and laboratory assistants. These staffs provide service to other patients in addition to pregnant women

Workload was calculated based on antenatal clinic attendance for the year 2014 using the following formula:

$$ W\rho =Y\rho /Z $$

Where *Wρ* is the average number of antenatal clients seen by each clinical staff per month in 2014 in a facility; *Yρ* is the total number of antenatal clients seen per month in 2014 in a facility and Z is the total number of antenatal clinical staffs in a facility.

Workload scores were calculated using the formula:

$$ S\rho i = W\rho i/W\rho j $$

Where *Sρi* represents the workload score for antenatal service in a specific facility (index facility); *Wρi* is the Workload per antenatal clinical staff in the index facility and *Wρj* is the highest workload per clinical staff amongst the 11 facilities (reference facility).

### Data analysis

Adherence to guidelines was computed by calculating the proportion of women whose first ANC visits met the criteria for complete adherence and corresponding confidence intervals. Adherence levels for the different types of facilities were compared at a significance level of *p = 0.05* using chi-square test. Facility factors were summarized using frequencies, ranges, means and standard deviations. Also, availability of human resource, antenatal drugs, laboratory testing and staff training on antenatal protocol was compared between the hospitals and polyclinics. Frequency of reported antenatal challenges was documented. Data entry and analysis were carried out using IBM SPSS Statistics for Windows, Version 20.0. Armonk, NY: IBM Corp

## Results

In all, 11 facilities from 11 districts and sub-metropolises were involved in the study that recruited 946 women between December, 2013 and May, 2014. There were six hospitals (54.5 %), 4 polyclinics (36.4 %) and one health center (9.1 %). The health center happens to be the primary care facility for the one of the districts without a hospital. With the exception of the health center, all the ten facilities were urban. All facilities offered ANC services everyday excluding weekends, except two polyclinics that offered the services 3–4 days in a week. A total of 63.6 % of facilities provided focused antenatal care and 18.2 % (one hospital and one polyclinic) providing a modified form of it. Focused antenatal care requires that a pregnant woman receives individualized care for a minimum of four ANC visits, with all services provided at the ANC unit. Some examples of modifications were that each client was seen by a midwife for history taking and examination, but some services like medication and laboratory testing were referred to another health worker outside the cubicle where the woman received consultation, or in some cases outside the maternity unit. Also, some facilities were not offering daily ANC services as prescribed by FANC and individualized counselling on danger signs were in some cases compromised for group talks for all ANC attendants.

All facilities indicated that antenatal care was provided by doctors and midwives (Table 1).

### Adherence to first antenatal care guidelines in facilities

Overall complete provider adherence to first antenatal care guidelines for all the participants was 48.1 % (95 % CI: 41.8–54.2 %), varying significantly amongst the individual facilities and the types of facilities. Complete provider adherence to guidelines was more common (51.9 %) in the polyclinics compared to the hospitals (47.8 %) and the only health center (33.8 %) (Table 2).

**Table 2:** Adherence levels for first antenatal care and antenatal workload score for the sampled facilities, in the Greater Accra Region, Ghana

Facility ID	Facility Type (N*)	Monthly antenatal attendants	Antenatal Workload score	Incomplete Adherence		Complete Adherence *N* (%)
				Poor *N* (%)	Moderate *N* (%)	
1	Ɨ Hospital (80)	624	0.11	12 (15.0)	26 (32.5)	42 (52.5)
2	Hospital (91)	1277	0.40	3 (3.3)	40 (44.0)	48 (52.7)
3	Hospital (102)	1894	0.57	19 (18.6)	44 (43.1)	39 (38.2)
4	Hospital (94)	1667	0.44	11 (11,7)	35 (37.2)	48 (51.1)
5	Hospital (79)	1631	0.72	26 (32.9)	18 (22.8)	35 (44.3)
6	Hospital (85)	1451	0.24	5 (5.9)	39 (45.9)	41 (48.2)
7	Polyclinic (90)	1.273	0.90	7 (7.8)	51 (56.7)	32 (35.5)
8	Polyclinic (85)	2563	1.00	9 (10.6)	25 (29.4)	51 (60.0)
9	Polyclinic (72)	1191	0.66	11 (15.3)	25 (34.7)	36 (50.0)
10	Polyclinic (94)	878	0.39	0 (0.0)	36 (38.3)	58 (61.7)
11	Health Center (74)	179	0.36	27 (36.5)	22 (36.5)	25 (33.8)
Total (*p* <*0.01*)				130 (13.7)	361 (38.2)	455 (48.1)
95 % Cl of mean						(41.8–54.2 %)

N* represents number of participants recruited in the facility; *CI* represents confidence interval

Ɨ The Regional hospital

### Human resource capacity of participating facilities

Antenatal clients seen per month in the health facilities ranged from 179 to 2563 women, with no significant difference between average numbers seen in hospitals and polyclinics. Total antenatal clinical staffs for the facilities ranged between nine (in the health center) and 119 (in a hospital) workers, with a mean (standard deviation) of 54.7 (32.5). The differences between the mean numbers of both clinical and support staffs in hospitals and polyclinics were statistically significant (*p* = 0.03). Average antenatal workload per month per clinical staff was higher in polyclinics compared to hospitals, resulting in relatively higher workload scores for the polyclinics (Table 3).

**Table 3:** Human Resource and logistics characteristics by facility type

Variable	Hospital	Polyclinic	*p*-value	All facilities	
	Mean (SD)	Mean (SD)		Range	Mean (SD)
Average monthly ANC attendance	1424.0 (181.2)	1476.3 (744.2)	0.89	179.0–2563.0	1329.8 (641.1)
ANC clinical staff^a^	75.0 (29.4)	35.8 (9.0)	0.03	9.0–119.0	54.7 (32.5)
Doctor/ OBGYN	18.7 (12.0)	3.6 (1.7)	0.02	1.0–40.0	11.8 (11.6)
Support staff^b^	22.7 (7.8)	11.8 (4.0)	0.03	6.0–36.0	17.2 (8.8)
ANC average workload per month^c^	22.7 (12.8)	41.4 (15.5)	0.07	6.2–55.7	29.2 (15.7)
ANC workload score	0.4 (0.2)	0.7 (0.3)	0.07	0.1–1.0	0.5 (0.3)
Availability of ANC drugs score	0.9 (0.1)	0.8 (0.1)	0.24	0.8–1.0	0.89 (0.13)
Availability of lab testing score	0.9 (0.1)	1.0 (0.0)	0.45	0.9–1.0	1.0 (0.0)
Proportion of clinical staff trained on SMP	0.4 (0.3)	0.7 (0.1)	0.17	0.2–0.0	0.6 (0.2)

*ANC* means antenatal care; *OBGYN* means obstetrician-gynecologist; *SD* means standard deviation; *SMP* means national safe motherhood service protocol

^a^ANC clinical staff refers to doctors (including specialists) and midwives

^b^ANC support staff refers to pharmacists, pharmacy technologists, dispensing technicians, biomedical scientists, laboratory technologists and laboratory assistants

^c^Unit of measurement: number of clients per health worker; p-values pertain to comparisons between the hospitals and polyclinics

### Availability of logistics, laboratory testing, equipment and protocols

There were no significant differences between hospitals and polyclinics with respect to availability of antenatal drugs, laboratory testing and trained on the SMP. Details on availability of essential drugs, laboratory tests, protocols and equipment for maternity care are shown in Table 4.

**Table 4:** Facilities with essential drugs, laboratory testing, protocols, equipment and newborn resuscitation capacity

Variable	Ambulance, blood and antenatal care drugs [*N* (%)]							
	Ambulance	Blood & blood products	Oxytocics	MgSO_4_	IV Fluids	Iron	SP for IPTp	ACT for Malaria
HC & PC (*N* = 5)	2 (40.0)	1 (20.0)	5 (100.0)	4 (80.0)	5 (100.0)	5 (100.0)	3 (60.0)	5 (100.0)
Hospitals (*N* = 6)	4 (66.7)	5 (83.3)	6 (100.0)	6 (100.0)	6 (100.0)	6 (100.0)	5 (66.7)	6 (100.0)
Variable	Laboratory testing [*n* (%)]							
	Glucose test	Malaria test	Hemoglobin test	Urine protein test	Ultrasound test	PMTCT for HIV	Pregnancy test	
HC & PC (*N* = 5)	5 (100.0)	5 (100.0)	5 (100.0)	5 (100.0)	*4 (80.0)* ^*3*^	5 (100.0)	5 (100.0)	
Hospitals (*N* = 6)	6 (100.0)	6 (100.0)	6 (100.0)	6 (100.0)	5 (83.3)	6 (100.0)	6 (100.0)	
Variable	Antenatal and delivery protocols and equipment [*n* (%)]							
	ANC protocols	Delivery protocols	SMP	STG	ANC equipment^a^	Delivery equipment^b^		
HC & PC (*N* 5)	5 (100.0)	5 (100.0)	5 (100.0)	5 (100.0)	5 (100.0)	5 (100.0)		
Hospital (*N* = 6)	6 (100.0)	6 (100.0)	6 (100.0)	6 (100.0)	6 (100.0)	6 (100.0)		
Variable	Newborn resuscitation [*n* (%)]							
	Vitamin K	Bag and mask	Heater or warmer	Incubator	Emergency tray	Oxygen supply		
HC & PC (*N* = 5)	5 (100.0)	5 (100.0)	*0 (0.0)*	*0 (0.0)*	5 (100.0)	5 (100.0)		
Hospital (*N* = 6)	5 (83.3)	6 (100.0)	4 (66.7)	3 (50.0)	6 (100.0)	6 (100.0)		

*ACT* means artemisinin based combination therapy; *ANC* means antenatal care; *HC* means health center; *PC* means polyclinic; *SMP* means national safe motherhood service protocol; *SP* means sulphadoxine-pyrimethamine; *STG* means Standard Treatment Guidelines

^a^weighing scale, sphygmomanometer, stethoscope, fetoscope, tape measure, thermometer, emergency tray

^b^weighing scale, sphygmomanometer, stethoscope, fetoscope, tape measure, thermometer, emergency tray, partograph, adequate lighting, adequate water source

A total of 6 (54.5 %) facilities had ambulances. Those without ambulances could call the National Ambulance Service (NAS) to transport clients when the need arises. One hospital had no blood transfusion service and referred patients who needed the service, in a similar manner as the polyclinics. Drugs such as iron preparations, artemisinin-based combination therapy for treatment of malaria, intravenous fluids and oxytocics were available in the pharmacies of all the facilities. Magnesium sulphate was found in all but one facility while sulphadoxine-pyrimethamine for intermittent presumptive treatment of malaria was available in only eight facilities.

All the facilities had laboratories that confirmed pregnancies and tested for blood glucose, malaria parasites, hemoglobin and urine protein, although availability of these tests at the antenatal unit of the facility varied. All facilities provided prevention of mother-to-child transmission (PMTCT) of human immune-deficiency virus (HIV) services which included both counseling and testing for HIV during antenatal care. One hospital had no ultrasound facility and clients had to do their scans elsewhere.

All 11 facilities had copies of antenatal and delivery service protocols, guidelines and wall charts, including the SMP and the national standard treatment guidelines (STG). Varying proportions of current health workers had been trained on the use of the SMP. All facilities had required antenatal and delivery equipment, including partograph for monitoring deliveries.

### Challenges with antenatal service delivery

All 11 facilities had challenges related to antenatal service delivery (Table 5). Inadequate numbers of staffs, especially midwives and inadequate operational space (36.4 % of facilities) were the prominent challenges expressed by facilities. Other challenges included lack of theatre and ambulance service, broken down equipment and poor inter-district and inter-facility collaboration in service delivery.

**Table 5:** List of antenatal and delivery problems as expressed by managers of sampled facilities in the Greater Accra Region, Ghana

Antenatal service delivery challenges	Health centre	Polyclinic	Hospital	Total
	*N*	*N*	*N*	*N* (%)
• Inadequate staffs, especially midwives	1	2	2	5 (45.5)
• Inadequate operational space	1	2	2	5 (45.5)
• Lack of theatre and ambulance services	0	1	1	2 (28.2)
• High attendance rate	0	1	0	1 (9.1)
• High rate of referrals out of the facility for varied reasons	0	1	0	1 (9.1)
• Broken down equipment	0	0	1	1 (9.1)
• Lack of blood transfusion services	0	0	1	1 (9.1)
• Irregular attendance	0	0	1	1 (9.1)
• Poor inter-district and inter-facility collaboration	0	0	1	1 (9.1)
• Poor public health interventions to support facility work	0	0	1	1 (9.1)

## Discussion

This paper reports on the availability of resources at different levels of health facilities in a low resource setting in relation to provider adherence to first antenatal visit guidelines within the facilities.

We do not consider our results to be totally representative of all first antenatal visits in the region especially in private facilities. This is because only data for women receiving antenatal care and delivering in public facilities was used, limiting the generalizability of the results to private practice. As mentioned earlier, most (62 %) facility based deliveries in the region occur in the public sector. However, we believe that the strength of this study lies in the fact that our result reflects everyday provider practice because adherence was retrospectively measured. Also, a description of the availability of resources within the facility (especially those required for antenatal care) allows us to assess expectations of provider adherence to guidelines in context.

Complete provider adherence to guidelines from our study was undoubtedly low but similar to what other studies on adherence reported in Ghana [17, 18] as well as in some other sub-Saharan countries like Kenya [19]. Also, the significant variation across the different types of facilities depicts non-uniformity of antenatal care during the first visit. This disparity in quality of care is further confirmed by the fact that not all facilities were providing the recommended focused antenatal care which ensures quality of care. Each facility provided all the routine antenatal services, and so perhaps providers were certain that at least sometime during the antenatal period, the pregnant woman would receive all they required, irrespective of the timelines. However, timing of care has been found to be an important determinant of quality of care, as it reduces patient cost and morbidity [18, 20, 21].

The World Health Organization (WHO) recommends that antenatal care be provided by a skilled attendant [22, 23], which in Ghana refers to a doctor, midwife, nurse or a community health officer [13, 23]. It is encouraging to note that all our study participants received care from either doctors or midwives, as is the situation in some advanced settings [24]. One previous study also reported that about 86 % of rural antenatal attendants in Ghana receive care from either midwives or doctors, and that women receiving antenatal care from these categories of health workers were more likely to have skilled attendance at delivery and post-natal care [25]. It should be noted however, that in other parts of the country especially in rural areas, like many sub-Saharan African countries, ANC clients are also seen by other health workers apart from doctors and midwives.

We cannot make definitive conclusions to what extent human resource availability influences adherence to guidelines, although there were significant differences between human resource availability across the facility types, with lower numbers in the polyclinics. Such differences also existed with respect to average workload per staff with the polyclinic staff being more burdened. However, complete provider adherence was higher in the polyclinics compared to the hospitals and the health center. Although it seems counterintuitive that the providers in facilities with higher workload adhere better to guidelines, this may be related in part to higher delivery workload at hospitals given that the polyclinics refer complications (during antenatal period and delivery) to the hospitals. It would have been helpful if we had data that allows us to calculate a weighted provider workload as per the exact amount of time spent on antenatal service alone, but this information was not readily available to us. Challenges in health system resources and the need for stronger systems have been documented to exist in many sub-Saharan African countries [26], and some of these relate to human resource [27]. Human resource quantity and quality is important in managing the burden of maternal morbidity and mortality and efforts should be aimed at training more and retaining existing ones in the sub-region [27].

As no significant difference in availability of laboratory testing, protocols and drugs for antenatal care at the different levels of care was observed, it is unlikely that these factors could explain the differences in provider complete adherence. Thus, while it appears that the facilities used for our study are equally resourced with respect to logistics and supplies for antenatal care, there exists variability in provider adherence to the antenatal guidelines.

Guidelines developed through local consensus building and problem based approaches are more likely to benefit from provider adherence [28]. We believe that to further understand the factors that determine provider adherence to available guidelines, providers need to be engaged. This study did not elicit provider factors but some qualitative studies have identified factors unrelated to health facility factors as contributing to provider use of guidelines [17, 29]. These studies emphasize the process of development of guidelines to assure its applicability, health worker attitude towards continuing education as well as negative perceptions about using guidelines during patient consultations amongst others. More of such exploratory studies that engage both frontline providers and healthcare managers need to be conducted in our study setting and specifically in relation to maternal health. This will not only identify the gaps and barriers to provider adherence but also possible ways of improving upon provider adherence to guidelines.

## Conclusion

Although human resource capacity for antenatal services varies across different facility types in the Greater Accra region, other health facility factors that support implementation of first antenatal care guidelines are equally available in all the facilities. These factors therefore do not adequately account for the low and varying proportions of provider complete adherence to guidelines across facility types. Providers should be continually engaged for a better understanding of the barriers to their adherence to these guidelines.

## Abbreviations

ANC: Antenatal care or antenatal clinic
CHPS: Community-based Health Planning and Services
ERC: Ethical review Committee
FANC: Focused antenatal care
GHS: Ghana health service
HIV: Human immune-deficiency virus
IPTp: Intermittent presumptive treatment in pregnancy
NAS: National ambulance service
NHIS: National health insurance scheme
PMTCT: Prevention of mother-to-child transmission
SMP: National safe motherhood service protocol
STG: Standard treatment guidelines
WHO: World Health Organization
